# A Total Diet Replacement Weight Management Program for Difficult-to-Treat Asthma Associated With Obesity: A Randomized Controlled Feasibility Trial

**DOI:** 10.1016/j.chest.2023.01.015

**Published:** 2023-01-14

**Authors:** Varun Sharma, Helen Clare Ricketts, Louise McCombie, Naomi Brosnahan, Luisa Crawford, Lesley Slaughter, Anna Goodfellow, Femke Steffensen, Duncan S. Buchan, Rekha Chaudhuri, Michael E.J. Lean, Douglas C. Cowan

**Affiliations:** a Institute of Infection, Immunity and InflammationSchool of Medicine, Dentistry and Nursing, College of Medical, Veterinary & Life Sciences, University of Glasgow, Glasgow, Scotland; b Human Nutrition Unit, School of Medicine, Dentistry and Nursing, College of Medical, Veterinary & Life Sciences, University of Glasgow, Glasgow, Scotland; c Clinical Research Facility, Glasgow Royal Infirmary, School of Health and Life Sciences, University of the West of Scotland, Glasgow, Scotland; d Division of Sport and Exercise, School of Health and Life Sciences, University of the West of Scotland, Glasgow, Scotland; e Counterweight, Ltd., London, England

**Keywords:** asthma, difficult asthma, obesity, severe asthma, weight management

## Abstract

**Background:**

Obesity is often associated with uncontrolled, difficult-to-treat asthma and increased morbidity and mortality. Previous studies suggest that weight loss may improve asthma outcomes, but with heterogenous asthma populations studied and unclear consensus on the optimal method of weight management. The Counterweight-Plus Programme (CWP) for weight management is an evidence-based, dietitian-led total diet replacement (TDR) program.

**Research Question:**

Can use of the CWP compared with usual care (UC) improve asthma control and quality of life in patients with difficult-to-treat asthma and obesity?

**Study Design and Methods:**

We conducted a 1:1 (CWP to UC) randomized, controlled single-center trial in adults with difficult-to-treat asthma and BMI of ≥ 30 kg/m^2^. The CWP was a 12-week TDR phase (800 kcal/d low-energy formula) followed by stepwise food reintroduction and weight loss maintenance for up to 1 year. The primary outcome was the change in Asthma Control Questionnaire 6 (ACQ6) score over 16 weeks. The secondary outcome was change in Asthma Quality of Life Questionnaire (AQLQ) score.

**Results:**

Thirty-five participants were randomized (36 screened) and 33 attended the 16-week follow-up (n = 17 in the CWP group, n = 16 in the UC group). Overall, mean ACQ6 score at baseline was 2.8 (95% CI, 2.4-3.1). Weight loss was greater in the CWP than UC group (mean difference, –12.1 kg; 95% CI, –16.9 to –7.4; *P* < .001). ACQ6 score improved more in the CWP than UC group (mean difference, –0.69; 95% CI, –1.37 to –0.01; *P* = .048). A larger proportion of participants achieved the minimal clinically important difference in ACQ6 score with CWP than with UC (53% vs 19%; *P* = .041; Number needed to treat, 3 [95% CI, 1.5-26.9]). AQLQ score improvement was greater in the CWP than UC group (mean difference, 0.76; 95% CI, 0.18-1.34; *P* = .013).

**Interpretation:**

Using a structured weight management program results in clinically important improvements in asthma control and quality of life over 16 weeks compared with UC in adults with difficult-to-treat asthma and obesity. This generalizable program is easy to deliver for this challenging phenotype. Longer-term outcomes continue to be studied.

**Trial Registry:**

ClinicalTrials.gov; No.: NCT03858608; URL: www.clinicaltrials.gov

## Graphical Abstract

**Figure:**
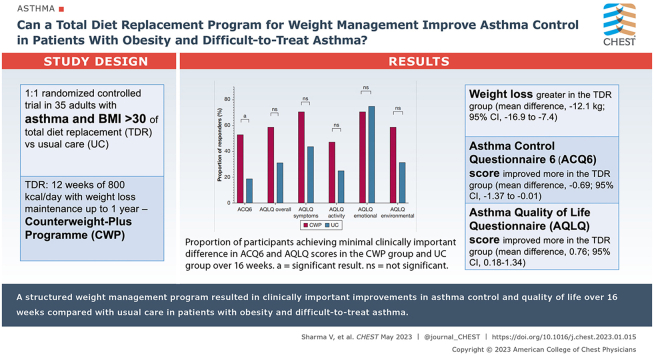

FOR EDITORIAL COMMENT, SEE PAGE 999

**Take-home Points**

**Study Question:** Can use of the Counterweight-Plus Programme (CWP) for weight management improve asthma control and quality of life in patients with difficult-to-treat asthma and obesity, compared with those receiving usual care (UC)?

**Results:** Over 16 weeks, the CWP resulted in clinically relevant improvements in both asthma control and quality-of-life indexes, with substantial weight loss, as compared with UC.

**Interpretation:** Initial results using the CWP are encouraging, and adherence to the program was better than expected, although longer-term outcomes are awaited to assess sustainability of the benefits seen.

Approximately 17% of people living with asthma have difficult-to-treat disease because of factors including poor inhaler technique, treatment nonadherence, and comorbidities such as obesity.^1^^,^^2^ Asthma associated with obesity is less steroid responsive; is linked with poorer control, quality of life, and increased morbidity and mortality; and has limited treatment options.^3^^,^^4^ The pathophysiologic effects of obesity on asthma are multifactorial. Weight excess has direct effects on thoracic wall mechanics,^5^ as well as increased airway closure,^6^^,^^7^ airway hyperresponsiveness,^8^^,^^9^ and airway inflammation.^10^, ^11^, ^12^ A Cochrane review^13^ of four studies (N = 197) suggested that weight loss may improve asthma control, but the quality of the evidence was poor and further well-constructed randomized controlled trials were recommended.

In the United Kingdom, the Counterweight-Plus Programme (CWP) for weight management is a commercially available dietitian-supported regimen of total diet replacement (TDR), stepwise food reintroduction, and weight loss maintenance. It has shown efficacy in obesity (mean weight loss, 10 kg; approximately one-third achieving loss of ≥ 15 kg) and type 2 diabetes mellitus (remission in 46% of patients).^14^^,^^15^ Its effects on asthma have not been evaluated, and we hypothesized that use of the CWP would result in improvements in asthma control and asthma-related quality of life. To test this hypothesis, we performed a randomized, controlled, proof-of-concept feasibility trial of CWP in patients with obesity and difficult-to-treat asthma. Herein, we report the primary outcome results for the first 16 weeks of treatment after completion of the first phase of the intervention program.

## Study Design and Methods

In this randomized, controlled, open-label, parallel study of a TDR weight loss program compared with usual care (UC) in individuals with difficult-to-treat asthma and obesity, participants were randomized 1:1 using a password-protected, online, third-party randomization service to CWP or UC.^16^ Study visits were scheduled at baseline and 16 weeks, with further visits planned for the 1-year and 2-year follow-up. The trial was approved by the West of Scotland Regional Ethics Committee (Identifier: 18/WS/0216), was sponsored and funded by an NHS Greater Glasgow and Clyde Endowment Fund, and is registered at ClinicalTrials.gov (Identifier: NCT03858608), where trial protocol is described.^17^ The funder and contributors to the fund had no input in study design or the trial outcomes. Because of the COVID-19 pandemic, face-to-face follow-up study visits were replaced with telephone consultations where necessary to optimize data collection. Recruitment and randomization was undertaken by the clinical research fellow. Study visits and data collection were performed by the clinical research fellow and clinical research nursing team at the Glasgow Royal Infirmary Clinical Research Facility.

### Participants

Eligible participants 18 to 75 years of age with BMI of ≥ 30.0 kg/m^2^, a diagnosis of asthma according to Global Initiative for Asthma guidelines,^18^ and difficult-to-treat disease according to Scottish Intercollegiate Guidelines Network/British Thoracic Society guidelines^19^ were identified from secondary and tertiary asthma clinics and ward admissions across NHS Greater Glasgow and Clyde (e-Appendix 1). Asthma clinicians and asthma specialist nurses referred patients to the clinical research fellow for screening after a brief explanation of the program. Asthma clinicians were aware of participation in the trial (consent forms were uploaded to electronic patient health-care records), but were not involved in recruitment, study visits, or data analysis. Eligible participants were provided with written information and were invited to attend the Glasgow Royal Infirmary Clinical Research Facility, where written informed consent was obtained before randomization and baseline data collection (visit 1). Participants were enrolled and randomized by the clinical research fellow.

### Measurements

Baseline demographics, asthma and other medical history, and medication information were obtained at visit 1. At all visits, the Asthma Control Questionnaire 6 (ACQ6) and Asthma Quality of Life Questionnaire (AQLQ) scores were recorded. The ACQ6 is a validated asthma control score comprising six questions,^20^ a score of ≥ 1.5 reflecting poor disease control, and with a minimal clinically important difference (MCID) of 0.5. The AQLQ is a validated score comprising 32 questions covering several domains (symptoms, activity limitation, emotional function, and environmental stimuli) assessing quality of life in asthma.^21^ A higher score reflects better quality of life and the MCID is 0.5.

At all visits, other data collected included anthropomorphic measures, health-care use, Medical Research Council dyspnea scale score, Hospital Anxiety Depression scale score, blood sampling, spirometry (Vitalograph ALPHA spirometer) as per European Respiratory Society/American Thoracic Society standards,^22^ peak expiratory flow rate, fractional exhaled nitric oxide (Feno; NIOX VERO; Aerocrine AB) according to American Thoracic Society guidelines,^23^ 6-min walk test according to European Respiratory Society/American Thoracic Society standards,^24^ and accelerometery (e-Appendix 1).

### Counterweight-Plus Program

The CWP consisted of three phases: TDR (0-12 weeks), food reintroduction (13-18 weeks), and weight loss maintenance (19-52 weeks) and was delivered by experienced dietitians with CWP training (e-Appendix 1). The TDR phase comprised a low-energy liquid diet consisting of 825 to 853 kcal/d (approximately 59% carbohydrate, 13% fat, 26% protein, and 2% fiber) administered via sachets of dried soups and shakes in a variety of flavors made up with water by the participant. The dietitian team reviewed participants at 1 week and then fortnightly. To allow flexibility for participants, acknowledging other commitments or logistical limitations, this phase was extended to 20 weeks if participants did not lose > 15 kg by week 12. Conversely, if a participant’s BMI fell to < 23.0 kg/m^2^, then food reintroduction was introduced earlier. The food reintroduction phase involved a reducing formula diet and stepwise reintroduction of calorie-controlled meals (with fortnightly dietitian review continuing). Flexible periods of 2 to 8 weeks were used for this phase based on participant confidence with weight loss management. In the weight loss maintenance phase, dietitians provided individually tailored calorie prescription for weight stabilization and to prevent weight regain, with monthly reviews. All program phases were underpinned by recognized behavior change strategies.^25^^,^^26^ Dietitian-led relapse treatments to correct weight regain were available.^27^

### Usual Care

Standard asthma care was continued in all participants in all groups. This included continuation of previously initiated asthma medication, but also modification of asthma treatment based on clinical need; those with worsening asthma received treatment escalation, whereas those with improving disease or lack of treatment efficacy underwent medication removal. All participants continued to be reviewed at their original secondary asthma clinic as part of standard care. All participants had the opportunity for weight management advice (ie, healthy eating and promoting exercise if in the UC group), inhaler technique, and asthma education as needed at each study visit.

### Primary Outcome

The primary outcome was difference in change in ACQ6 score from baseline (visit 1) to 16 weeks (visit 2) between the CWP and UC groups.

### Secondary Outcomes

Secondary measures included difference in change in AQLQ score from baseline to 16 weeks between CWP and UC groups, overall and in each AQLQ domain (symptoms, activity, emotional, and environmental), and the difference in proportion of participants with ≥ 0.5 change (MCID in ACQ6^20^ and AQLQ^21^) between groups at 16 weeks. For other outcomes, see e-Appendix 1.

### Sample Size

To demonstrate a difference of 0.5 between mean changes in ACQ6 score in CWP and UC groups from baseline to 16 weeks, based on an SD of 0.5 from a similar population,^28^ a sample size of 30 (15 per group) was required, assuming an α values of 0.05, a β values of 0.2, and power of 0.8. A target of 40 participants was chosen to allow for a 25% dropout rate.

### Statistical Analysis

Participants attending visits 1 and 2 were included for intention-to-treat analysis. Continuous variables were described as mean (95% CI) or median (interquartile range [IQR]) based on distribution and compared using independent *t* tests or Mann-Whitney *U* tests, respectively. Change in continuous variables over time was analyzed using analysis of covariance with the baseline variable as a covariate and comparing change in variables using *t* tests or Mann-Whitney *U* tests, depending on distribution. Categorical variables were described as number (percentage) and were compared using Pearson χ ^2^ test or the Fisher exact test as appropriate. Analyses were performed using IBM SPSS Statistics for Mac version 28 (IBM Corp.); graphs were produced using GraphPad Prism for Mac version 9.3.1 (GraphPad Software). A *P* value of ≤ .05 was significant. All data analysis was performed by the clinical research fellow using anonymized data.

## Results

### Participation and Baseline Characteristics

**Figure 1:**
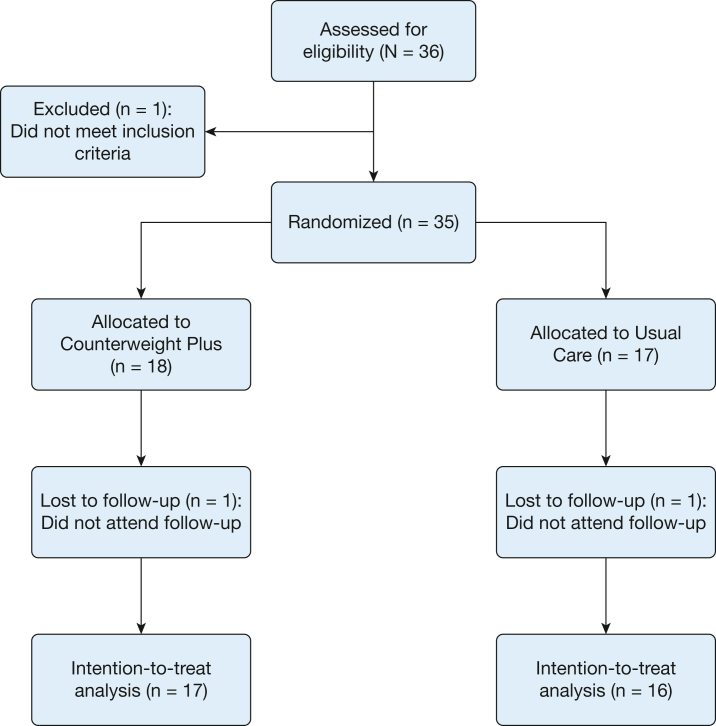
Consolidated Standards of Reporting Trials flow chart.

Participants were recruited from August 2019 through August 2021, with 2-year follow-up scheduled to finish in August 2023. Sixteen-week follow-up visits continued until December 2021. Of 36 participants screened, one was ineligible (e-Appendix 1) and 35 were randomized. Two patients were lost to follow-up and 33 patients attended visit 2 to be included in the intention-to-treat analysis (n = 17 in the CWP group, n = 16 in the UC group; Fig 1). Recruitment was halted before the target of 40 because of a lower than expected dropout rate.

Overall, mean age was 53 years, 63% were female sex, 54% were former smokers, and 43% were never smokers (Table 1). Comorbidities were common, including atopy (71%), allergic (54%) and perennial (46%) rhinitis, gastroesophageal reflux disease (86%), mental health problems (51%), and osteopenia or osteoporosis (43%). Significant treatment burden was observed, notably with 17% taking maintenance prednisolone and just over one-third receiving biological treatment. The study population consisted of those with frequent exacerbations with uncontrolled disease as reflected by the median for oral corticosteroid courses in the previous 12 months of 3 (IQR, 2-5) and mean ACQ6 score of 2.8 (95% CI, 2.4-3.1). Mean overall AQLQ score was 3.8 (95% CI, 3.4-4.2). Median weight was 101.7 kg (IQR, 91.4-118.7 kg), with a median BMI of 37.5 kg/m^2^ (IQR, 35.0-42.3 kg/m^2^), mean waist to hip ratio of 0.99 and mean waist to height ratio of 0.74, all suggestive of a morbidly obese, high-risk population (e-Table 1). Low median Feno and eosinophil count (18 ppb and 0.11 × 10^9^/L, respectively) suggested predominance of a Type 2 (T2)-low endotype within the population.

**Table 1:** Baseline Characteristics

Variable	Overall (n = 35)	CWP (n = 18)	UC (n = 17)
Age, y	52.6 (48.3-56.9)	56.7 (51.3-62.1)	48.3 (41.5-55.1)
Female sex	22 (62.9)	13 (72.2)	9 (52.9)
Smoking status			
Current smoker	1 (2.9)	0 (0.0)	1 (5.9)
Former smoker	19 (54.3)	12 (66.7)	7 (41.2)
Lifelong nonsmoker	15 (42.9)	6 (33.3)	9 (52.9)
Smoking, pack-years∗	15.0 (6.0-30.0)	15.0 (5.0-22.5)	5.0 (0.0-20.0)
Age at asthma diagnosis, y	30.9 (23.8-38.1)	34.3 (24.1-44.4)	27.4 (16.6-38.2)
Duration of asthma, y	21.7 (16.5-27.0)	22.5 (13.7-31.3)	20.9 (14.3-27.5)
Atopy	25 (71.4)	12 (66.7)	13 (76.5)
Allergic rhinitis	19 (54.3)	9 (50.0)	10 (58.8)
Perennial rhinitis	16 (45.7)	7 (38.9)	9 (52.9)
Nasal polyps	4 (11.4)	3 (16.7)	1 (5.9)
Nasal surgery	4 (11.4)	3 (16.7)	1 (5.9)
Eczema	13 (37.1)	6 (33.3)	7 (41.2)
GERD	30 (85.7)	16 (88.9)	14 (82.4)
ILO/DFB	8 (22.9)	5 (27.8)	3 (17.6)
Psychological illness	18 (51.4)	8 (44.4)	10 (58.8)
Emphysema	5 (14.3)	3 (16.7)	2 (11.8)
Bronchiectasis	1 (2.9)	1 (5.6)	0 (0.0)
SAFS/ABPA	9 (25.7)	3 (16.7)	6 (35.3)
Diabetes mellitus	4 (11.4)	4 (22.2)	0 (0.0)
Hypertension	9 (25.7)	6 (33.3)	3 (17.6)
Cardiac disease	7 (20.0)	2 (11.1)	5 (29.4)
Osteopenia/osteoporosis	15 (42.9)	6 (33.3)	9 (52.9)
BDP equivalent dose, μg∗	1,600 (1,600-2,000)	1,600 (1,600-1,600)	2,000 (1,600-2,400)
LAMA	33 (94.3)	18 (100.0)	15 (88.2)
Maintenance prednisolone	6 (17.1)	4 (22.2)	2 (11.8)
Prednisolone dose, mg	4.5 (1.2-7.8)	4.5 (–1.9 to 10.9)	4.5 (–1.9 to 10.9)
Montelukast	27 (77.1)	14 (77.8)	13 (76.5)
Theophylline	22 (62.9)	10 (55.6)	12 (70.6)
Azithromycin	7 (20.0)	6 (33.3)	1 (5.9)
Omalizumab	4 (11.4)	1 (5.6)	3 (17.6)
Mepolizumab	8 (22.9)	4 (22.2)	4 (23.5)
Antihistamine	24 (68.6)	11 (61.1)	13 (76.5)
Nasal steroid	24 (68.6)	12 (66.7)	12 (70.6)
PPI/H2A	30 (85.7)	17 (94.4)	13 (76.5)
Previous 12 mo∗			
Prednisolone courses	3 (2-5)	4 (2-5)	3 (2-5)
Out-of-hours GP visit	0 (0-0)	0 (0-0)	0 (0-0)
ED visit	0 (0-0)	0 (0-0)	0 (0-0)
Hospital admissions	0 (0-1)	0 (0-0)	0 (0-1)
ICU admissions	0 (0-0)	0 (0-0)	0 (0-0)
Weight, kg∗	101.7 (91.4-118.7)	103.3 (96.9-118.3)	97.0 (86.5-122.0)
BMI, kg/m^2^^∗^	37.5 (35.0-42.3)	38.2 (35.6-45.3)	36.1 (32.7-42.5)
MRC dyspnea scale score∗	3 (3-4)	3 (3-4)	3 (3-4)
ACQ6 score	2.8 (2.4-3.1)	2.8 (2.2-3.3)	2.8 (2.2-3.3)
AQLQ score			
Overall	3.8 (3.4-4.2)	3.8 (3.3-4.4)	3.8 (3.2-4.4)
Symptom domain	3.8 (3.4-4.2)	3.7 (3.2-4.3)	3.8 (3.2-4.5)
Activity domain	3.8 (3.4-4.2)	3.9 (3.4-4.4)	3.7 (3.0-4.3)
Emotional domain	3.8 (3.2-4.3)	3.6 (2.8-4.5)	3.9 (3.1-4.7)
Environmental domain	4.1 (3.6-4.6)	4.0 (3.4-4.6)	4.2 (3.4-5.0)
HAD score∗			
Anxiety scale	8 (6-11)	9 (7-11)	7 (5-11)
Depression scale	8 (5-11)	8 (5-11)	9 (7-14)
Eosinophils, × 10^9^/L∗	0.11 (0.08-0.42)	0.17 (0.08-0.42)	0.1 (0.04-0.51)
Feno, ppb∗	18 (11-33)	15 (10-35)	20 (13-51)
PEF, L/min	375 (334-415)	318 (275-360)	435 (374-496)
Spirometry, %			
FEV_1_ before BD administration	72.1 (66.0-78.1)	65.8 (57.1-74.6)	78.7 (70.7-86.7)
FEV_1_ to FVC ratio before BD administration	70.4 (67.2-73.5)	67.9 (62.5-73.2)	73.0 (69.7-76.2)
FEV_1_ change after BD administration	3.4 (1.3-5.4)	5.1 (1.5-8.7)	1.5 (-0.5, 3.6)
6MWD, m	326 (284-367)	315 (250-381)	337 (282-393)

Data are presented as No. (%), mean (95% CI) if parametric, or median (interquartile range) if nonparametric (the latter denoted by ∗). 6MWD = 6-min walk distance; ABPA = Allergic Bronchopulmonary Aspergillosis; ACQ6 = Asthma Control Questionnaire 6; AQLQ = Asthma Quality of Life Questionnaire; BD = bronchodilator; BDP = beclomethasone dipropionate; CWP = Counterweight-Plus Programme; DFB = dysfunctional breathing; Feno = fractional exhaled nitric oxide; GERD = gastroesophageal reflux disease; GP = general practitioner; HAD = Hospital Anxiety and Depression; H2A = H2-receptor antagonist; ILO = inducible laryngeal obstruction; LAMA = long-acting antimuscarinic; MRC = Medical Research Council; PEF = peak expiratory flow; ppb = parts per billion; PPI = proton pump inhibitor; SAFS = severe asthma with fungal sensitization; UC = usual care.

Individuals in the CWP group were slightly older, had lower baseline peak expiratory flow rate and FEV_1_, and were more sedentary, with accelerometery data demonstrating more inactive time and less time spent in light to moderate vigorous physical activity compared with the UC group. No other between-group differences were observed.

### Primary Outcome

**Figure 2:**
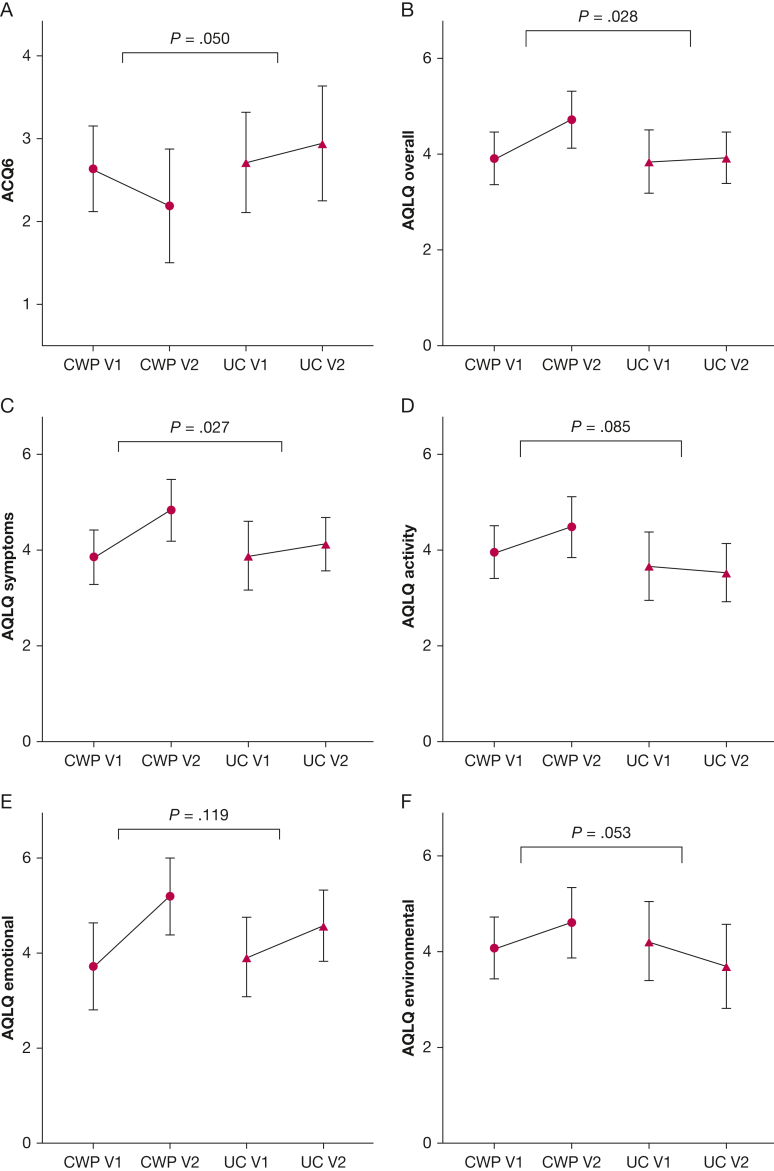
A-F, Graphs showing change in ACQ6 score (A), AQLQ overall score (B), AQLQ symptom domain score (C), AQLQ activity domain score (D), AQLQ emotional domain score (E), and AQLQ environmental domain score (F) between the CWP group and UC group at baseline (V1) and 16 weeks (V2). P value compares change in variable between CWP and UC groups with independent t test. ACQ6 = Asthma Control Questionnaire 6; AQLQ = Asthma Quality of Life Questionnaire; CWP = Counterweight-Plus Program; UC = usual care.

Over 16 weeks, mean change in ACQ6 score was –0.45 (95% CI, –1.02 to 0.13) for the CWP group and 0.23 (95% CI, –0.17 to 0.63) for the UC group, with a mean difference of –0.69 (95% CI, –1.37 to –0.01; *P* = .048) between groups (Table 2, e-Table 2, Fig 2).

**Table 2:** Intention-to-Treat Comparison of Asthma Control and Quality-of-Life Outcomes Between CWP and UC Groups Over 16 Weeks

Variable	CWP Group (n = 17)	UC Group (n = 16)	Mean Difference Between CWP and UC Groups	*P* Value^a^
ACQ6	–0.45 (–1.02 to 0.13)	0.23 (–0.17 to 0.63)	–0.69 (–1.37 to –0.01)	.048
AQLQ				
Overall	0.81 (0.28-1.35)	0.08 (–0.32 to 0.48)	0.76 (0.18-1.34)	.013
Symptom domain	0.98 (0.44-1.52)	0.25 (–0.13 to 0.63)	0.72 (0.14-1.31)	.018
Activity domain	0.53 (0.01-1.05)	–0.13 (–0.73 to 0.46)	0.78 (0.08-1.47)	.029
Emotional domain	1.47 (0.59-2.35)	0.66 (0.07-1.25)	0.72 (–0.16 to 1.59)	.104
Environmental domain	0.52 (–0.26 to 1.30)	–0.52 (–1.30 to 0.26)	0.98 (0.01-1.96)	.048

Data are presented as mean (95% CI). ACQ6 = Asthma Control Questionnaire 6; AQLQ = Asthma Quality of Life Questionnaire; CWP = Counterweight-Plus Programme; UC = usual care.

a Comparison of mean difference using analysis of covariance with baseline variable as covariate.

### Secondary Outcomes

**Figure 3:**
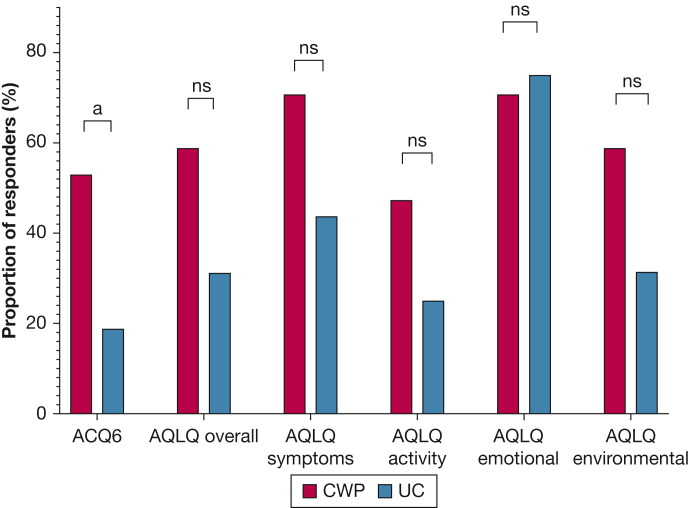
Bar graph showing the proportion of participants achieving minimal clinically important difference in ACQ6 and AQLQ scores in the Counterweight-Plus Program group and usual care group over 16 weeks. Compared using χ ^2^ or Fisher exact test. ^a^Significant result. ACQ6 = Asthma Control Questionnaire 6; AQLQ = Asthma Quality of Life Questionnaire; ns = not significant*.*

Over 16 weeks, mean change in overall AQLQ score was 0.81 (95% CI, 0.28-1.35) for the CWP group and 0.08 (95% CI, –0.32 to 0.48) for the UC group, with a mean difference of 0.76 (95% CI, 0.18-1.34; *P* = .013) between groups (Table 2, Fig 2). Likewise, mean changes in AQLQ symptom, activity, and environmental domain scores favored the CWP group, with mean between-group differences of 0.72 (95% CI, 0.14-1.31; *P* = .018), 0.78 (95% CI, 0.08-1.47; *P* = .029), and 0.98 (95% CI, 0.01-1.96; *P* = .048), respectively. Change in AQLQ emotional domain score was not significantly different between groups. A greater proportion of participants achieved ACQ6 score MCID with CWP compared with UC (53% vs 19%, respectively; *P* = .041; NNT = 3 [95% CI, 1.5-26.9]), but no significant differences were seen for proportions achieving MCID for AQLQ score overall or within the four AQLQ domains (Fig 3, e-Table 3). No changes were observed in number of prednisolone courses, out-of-hours general practitioner or ED visits, hospital admissions, or ICU admissions between the two groups.

### Other Outcomes

Mean weight loss was –13.5 kg (95% CI, –17.5 to –9.6) for the CWP group and –1.4 kg (95% CI, –3.2 to 0.4) for the UC group (mean difference, –12.1 kg; 95% CI, –16.9 to –7.4 kg; *P* < .001), with a mean total body weight loss of approximately 12% with CWP (Table 3). BMI change was –4.9 kg/m^2^ (95% CI, –6.3 to –3.5 kg/m^2^) for CWP and –0.3 kg/m^2^ (95% CI, –1.1 to 0.6 kg/m^2^) for UC (mean difference, –4.6 kg/m^2^ [95% CI, –6.3 to –2.9 kg/m^2^]; *P* < .001). Median change in Medical Research Council dyspnea scale score was –1 (IQR, –1 to 0) for CWP and 0 (IQR, 0-0) for UC (*P* = .004). No significant between-group differences were found for spirometry, 6-min walk distance, Borg breathlessness scale score, Hospital Anxiety and Depression scale scores, peripheral eosinophil counts, Feno, or accelerometery (e-Table 4).

**Table 3:** Intention-to-Treat Comparison of Other Outcomes Between CWP and UC Groups

Variable	CWP Group		UC Group		Mean Difference Between CWP and UC	*P* Value^a^
	No.	Difference	No.	Difference		
Weight, kg	13	–13.5 (–17.5 to –9.6)	9	–1.4 (–3.2 to 0.4)	–12.1 (–16.9 to –7.4)	< .001
Total body weight, %	13	–12.3 (–15.7 to –8.8)	9	–1.2 (–3.0 to 0.7)	–11.1 (–15.4 to –6.9)	< .001
BMI, kg/m^2^	13	–4.9 (–6.3 to –3.5)	9	–0.3 (–1.1 to 0.6)	–4.6 (–6.3 to –2.9)	< .001
MRC dyspnea scale score∗	16	–1 (–1 to 0)	15	0 (0-0)	N/A	.004
HAD score						
Anxiety scale	17	1 (–1 to 3)	16	1 (–1 to 2)	0 (–3 to 3)	.972
Depression scale	17	–1 (–3 to 2)	16	1 (–1 to 2)	–1 (–4 to 2)	.445
Eosinophil count, × 10^9^/L∗	8	0.05 (0.00-0.11)	6	0.00 (–0.23 to 0.12)	N/A	.228
Feno, ppb∗	8	1 (–3 to 21)	6	–6 (–28 to 18)	N/A	.573
PEF, L/min	9	38 (–16 to 91)	6	7 (–36 to 49)	31 (–37 to 99)	.343
Spirometry						
FEV_1_ before BD administration, %	8	5.5 (–3.2 to 14.2)	6	3.7 (–1.4 to 8.8)	1.8 (–7.5 to 11.1)	.671
FEV_1_ to FVC ratio before BD administration	8	–1.96 (–4.23 to 0.32)	6	1.09 (–4.25 to 6.43)	–3.0 (–8.4 to 2.3)	.224
FEV_1_ after BD administration, %	8	3.4 (–2.8 to 9.6)	6	4.2 (–7.3 to 15.7)	–0.8 (–11.5 to 9.9)	.874
Annualized health-care use∗						
Prednisolone courses	17	–2 (–2 to 0)	16	–2 (–3 to 1)	N/A	.790
Out-of-hours GP visits	17	0 (0-3)	16	0 (0-3)	N/A	.737
ED visits	17	0 (0-0)	16	0 (0-0)	N/A	.557
Hospital admissions	17	0 (0-0)	16	0 (–1 to 0)	N/A	.510
ICU admissions	17	0 (0-0)	16	0 (0-0)	N/A	1.000
6MWD, m	8	8 (–16 to 31)	5	0 (–50 to 50)	8 (–34 to 49)	.698

Data are presented as mean (95% CI) for parametric data or median (interquartile range) for nonparametric data (the latter denoted by ∗). Annualized health-care use variables compare change from baseline data (No. of events in prior 12 mo) to 16 wk ([No. of events × 365] / No. of d between visits). 6MWD = 6-min walk distance; BD = bronchodilator; CWP = Counterweight-Plus Programme; Feno = fractional exhaled nitric oxide; GP = general practitioner; HAD = Hospital Anxiety and Depression; MRC = Medical Research Council; N/A = not available; PEF = peak expiratory flow; ppb = parts per billion; UC = usual care.

a Comparison using independent *t* test for parametric data or Mann-Whitney *U* test for nonparametric data.

### Per-Protocol Analysis

Of the 33 participants attending visit 2, two participants did not tolerate CWP (e-Appendix 1). Thirty-one participants were included for per-protocol analysis. Mean weight loss was greater in the CWP group than the UC group (mean difference, –13.3 kg; 95% CI, –17.2 to –9.4; *P* < .001), with a 13% loss of total body weight with CWP (e-Table 6). Over 16 weeks, mean change in ACQ6 score was –0.60 (95% CI, –1.20 to 0.01) for CWP and 0.23 (95% CI, –0.17 to 0.63) for UC (mean difference, –0.86 [95% CI, –1.55 to –0.18]; *P* = .015; e-Table 5). Mean change in overall AQLQ score was 0.97 (95% CI, 0.42-1.53) for CWP and 0.08 (95% CI, –0.32 to 0.48) for UC (mean difference, 0.95 [95% CI, 0.40-1.50]; *P* = .001). Likewise, mean changes in AQLQ symptom, activity, and environmental domain scores favored CWP with mean between-group differences of 0.89 (95% CI, 0.32-1.46; *P* = .003), 0.97 (95% CI, 0.32-1.62; *P* = .005), and 1.18 (95% CI, 0.21-2.14; *P* = .018), respectively. Change in AQLQ emotional domain score was not significantly different between groups. A greater proportion of participants achieved MCID for ACQ6 and AQLQ scores with CWP compared with UC (ACQ6, 60% vs 19% [*P* = .018]; AQLQ, 67% vs 31% [*P* = .049]; e-Table 7). No significant between-group differences were found for separate AQLQ domains.

### Weight Loss Extent and Change in ACQ6 and AQLQ Scores

Post hoc analysis of changes in ACQ6 and AQLQ scores with CWP in groups based on extent of total body weight loss (< 10%, 10%-15%, and ≥ 15%) showed trends toward greater benefit with greater weight loss (Table 4). Within 10% to 15% and ≥ 15% weight loss groups, mean ACQ6 score change was –0.7 (95% CI, –1.6 to 0.3) and –1.2 (95% CI, –3.1 to 0.7), respectively, and mean change in AQLQ score was 0.6 (95% CI, –0.1 to 1.3) and 1.4 (95% CI, –0.8 to 3.6), respectively. Similar trends were seen for each of the four AQLQ domains.

**Table 4:** Post Hoc Comparison of Asthma Control and Quality of Life With CWP by Percentage Weight Loss

Variable	< 10% Group (n = 3)	10%-15% Group (n = 6)	≥ 15% Group (n = 4)	*P* Value^a^
ACQ6 score	–0.1 (–2.0 to 1.8)	–0.7 (–1.6 to 0.3)	–1.2 (–3.1 to 0.7)	.390
AQLQ score				
Overall	0.2 (–2.1 to 2.5)	0.6 (–0.1 to 1.3)	1.4 (–0.8 to 3.6)	.309
Symptom domain	0.5 (–1.1 to 2.0)	0.9 (0.1-1.8)	1.7 (–0.4 to 3.9)	.259
Activity domain	–0.1 (–4.2 to 4.0)	0.4 (–0.3 to 1.0)	1.3 (–0.5 to 3.1)	.236
Emotional domain	0.8 (–2.7 to 4.3)	1.0 (–0.4 to 2.4)	1.4 (–1.8 to 4.6)	.876
Environmental domain	–0.4 (–3.8 to 3.0)	0.3 (–1.7 to 2.3)	0.9 (–1.2 to 2.9)	.625

Data are presented as mean (95% CI). ACQ6 = Asthma Control Questionnaire 6; AQLQ = Asthma Quality of Life Questionnaire; CWP = Counterweight-Plus Programme.

a Comparison of mean difference using analysis of variance.

### Adverse Events

No unexpected serious adverse events or intervention-related adverse events occurred during the trial. Overall, five participants were hospitalized during the 16-week period: three participants in the UC group (one participant with a ward level exacerbation of asthma, one participant with exacerbation of asthma requiring high dependency monitoring, one participant with COVID-19 pneumonitis) and two participants in the CWP group (one participant with COVID-19 gastroenteritis and one participant with migraine).

## Discussion

In this pragmatic open-label, randomized, controlled trial, we showed that delivery of a supported low-calorie total diet replacement program (Counterweight-Plus) to patients with difficult-to-treat asthma and obesity was safe and led to significant improvements in asthma control and quality of life compared with UC over 16 weeks. We demonstrated clinically significant improvements in favor of CWP for ACQ6 score, AQLQ score overall, and symptoms, activity, and environmental AQLQ domains. Comparison by percentage total body weight loss showed that > 10% loss is needed to gain clinically relevant benefits, although loss of > 15% likely imparts greater benefit. In addition, CWP showed favorable impacts on exertional breathlessness and anthropometric measures, the latter likely to have important consequences for other aspects of general health. These findings suggest that conservative treatment targeting substantial weight loss in patients with difficult-to-treat asthma and obesity is safe and can impact patient-centered outcomes favorably. Longer-term outcomes are awaited to determine whether benefits persist. This program can be administered in a primary care setting.

A small number of trials have evaluated the impact of weight loss interventions in the population with obesity-associated asthma, with varying methodologies and outcomes. Freitas et al^29^ reported improvements in ACQ6 and AQLQ scores in a randomized trial of cardiovascular exercise for 3 months compared with sham breathing and stretching in asthma (n = 51). Weight loss was lower than in the present study (6 kg), the population studied differed at baseline considerably (predominantly female sex [98%], lower weight [91 kg], higher eosinophil level [> 0.3 × 10^9^/L], and lower ACQ6 score [2.0]), the definition and criteria for disease severity were not prespecified, and participants taking daily oral corticosteroids were excluded. Trial pragmatism and generalizability are unclear because of the 645 participants screened, only 55 participants were eligible (167 participants had no documented reason for exclusion).

Conversely, a study of 330 participants (of 2,022 screened) reported by Ma et al^30^ showed no significant change in ACQ6 score or quality of life with a lifestyle intervention protocol (calorie reduction, moderate-intensity physical activity, and behavioral self-management skills) compared with standard care. Baseline weight (104.2 kg) and BMI (37.5 kg/m^2^) were comparable with the present population, although baseline asthma control was markedly better (mean ACQ6 score, 1.4). Participants requiring daily oral corticosteroids were excluded. However, the mean weight loss was much lower than in the present trial (5 kg) at 6 months, probably insufficient to impact significantly on asthma-related outcomes. Subanalysis suggested improved ACQ6 score in those with weight loss of > 5% and larger effects with weight loss of > 10%.

Scott et al^28^ reported a randomized uncontrolled three-arm parallel trial of either dietary or exercise intervention or both over 10 weeks in participants with BMI of 28 to 40 kg/m^2^ and asthma. Per-protocol analysis showed mean ± SD weight loss was lower than in the present trial (8.5 ± 4.2%, 1.8 ± 2.6%, and 8.3 ± 4.9% with diet, exercise, and combined interventions, respectively), with improved mean ACQ6 score in the diet and combined groups (–0.6 ± 0.5 and –0.5 ± 0.7, respectively) and median AQLQ score in all groups (0.9 [IQR, 0.4-1.3], 0.49 [IQR, 0.03-0.78], and 0.5 [IQR, 0.1-1.0] for the diet, exercise, and combined groups, respectively). However, as well as lacking a control group, the population showed better control (ACQ6 score range, 1.00-1.36), better quality of life (AQLQ score range, 5.8-6.8), and lower inahled corticosteroid doses (1,000 μg beclomethasone dipropionate equivalent) at baseline compared with the present difficult-to-treat population.

Özbey et al^31^ performed a randomized controlled trial (n = 55) of patients with asthma and BMI of ≥ 30 kg/m^2^ comparing standard care with a 10-week dietitian-led weight loss program. They reported improved asthma control and quality-of-life scores; however, they studied an almost entirely female (96%) general asthma population with uncertainty around the diagnosis of asthma, active disease, and disease severity. Furthermore, the mean Asthma Control Test score of 21 suggests a population whose disease is well controlled. The authors correctly question the generalizability based on these limitations.

Grandi Silva et al^32^ reported a trial of weight loss (nutritional support, psychology input, and a varied exercise program) in women with BMI of 35 to 40 kg/m^2^ and moderate to severe asthma (n = 51) to assess the effect on dynamic hyperinflation. Post hoc analysis showed improvements in ACQ6 score and AQLQ score in those who lost ≥ 5% body weight, although no significant change in ACQ6 score was found when compared with those who lost < 5% body weight. The lack of randomization and a control group, as well as unclear details of the intervention and results are limitations.

This trial has several possible limitations. This proof-of-concept feasibility study was sufficient to detect significant effects, but a larger study is needed to generate definitive results. Small differences were observed between groups at baseline (age, peak expiratory flow rate, FEV_1_, and accelerometery), although because of randomization, these were unlikely to impact the primary and secondary outcomes we obtained. Baseline asthma control and quality-of-life measures (which are affected by factors such as lung function and activity levels) were similar in both groups, suggesting that potential clinical gains would be similar in both groups. National lockdowns during the COVID-19 pandemic limited data collection (39% complete datasets) for variables requiring physical attendance (specifically, blood tests, Feno, spirometry, 6-min walk test, and accelerometery), although data for primary and key secondary outcomes were complete (e-Appendix 1). With a higher proportion of complete datasets, it would be possible to assess differences in other key outcomes (eg, lung function and inflammation). As with all weight management studies, this was an open-label trial potentially subject to biases that may affect treatment effect estimates. This trial was conducted in a real-life clinical setting where asthma clinicians could be aware of the substantial effects on body weight from the intervention, and as such, masking would not have been feasible. It is feasible that participants pleased with weight loss in the intervention group, might have been included more often to minimize asthma symptoms, and thus would generate a more positive response to the intervention than their physiologic features might reveal. However, this still constitutes a positive beneficial outcome from both patient and health-care perspectives. Certain variables (eg, number of exacerbations) were reliant on participant recollection, and thus are subject to recall bias. Participants willing to take part in a weight loss trial are more likely to be motivated to lose weight, leading to potential selection bias, although this would not detract from clinical value. Key strengths of the trial include the pragmatic and real-world applicability of the intervention. Randomization led to broadly comparable CWP and UC groups, which adds confidence to reported results. The population studied is one with difficult-to-treat asthma with frequent exacerbations, an at-risk group with disease that is troublesome to manage.

Longer-term follow-up is required to determine whether weight loss is maintained and whether asthma-related benefits persist. Additionally, a future trial with a greater sample size is justified to generate definitive results. Further research should explore the factors associated with successful treatment outcome and efficacy in the overweight (BMI, 25.0-29.9 kg/m^2^) population with difficult-to-treat asthma.

## Interpretation

Compared with UC, use of the CWP weight management program with dietitian support improved asthma control and quality of life as well as dyspnea and anthropomorphic measures over 16 weeks in individuals with difficult-to-treat asthma and obesity. Further research is needed to confirm the longer-term outcomes and to identify predictors of treatment response.

## Funding/Support

This study was funded by an NHS Greater Glasgow and Clyde Endowment fund.

## Financial/Nonfinancial Disclosures

The authors have reported to *CHEST* the following: V. S. and H. C. R. have received travel awards to attend conferences. N. B. has received funding from Cambridge Weight Plan for PhD and conference and travel expenses and has shares in and is an employee of Counterweight Ltd. D. S. B. has received a CSO grant unrelated to this study. R. C. has received funding from 10.13039/100004325AstraZeneca for a study within a Medical Research Council project as investigator lead; has received payment for lectures from GSK, AstraZeneca, Teva, Chiesi, Sanofi, and Novartis; has received funding to attend conferences from Chiesi, Sanofi, and GSK; and has participated on advisory board meetings for GSK, AstraZeneca, Teva, Chiesi, and Novartis. M. E. J. L. has received grants from NIHR, 10.13039/501100000361Diabetes UK, 10.13039/501100000318All Saints Educational Trust, 10.13039/501100004191Novo Nordisk, and MJ Smith Trust for the study; has received consulting fees from Novo Nordisk and Nestle; has received payment for lectures from Oviva, Merck, Sanofi, and Roche; and is a medical advisor for Counterweight Ltd. None declared (L. M., L. C., L. S., A. G., F. S., D. C. C.).

## Supplementary Data

e-Online Data
