# Supplementary material for: A Total Diet Replacement Weight Management Program for Difficult-to-Treat Asthma Associated With Obesity: A Randomized Controlled Feasibility Trial
Source: Chest. 2023 Jan 14;163(5):1026–37. doi: 10.1016/j.chest.2023.01.015 (PMC10808069; doi:10.1016/j.chest.2023.01.015)
Supplement: e-Online Data [file mmc1.docx]

**Supplementary information**

**Methods**

*Participants*

Asthma diagnosis as per Global Initiative for Asthma guidelines [1] was confirmed with evidence of characteristic variable asthma symptoms and either airway reversibility or hyperresponsiveness in the preceding 5 years defined as: ≥200mls and 12% increase in forced expiratory volume in 1 second (FEV_1_) post-bronchodilator or after ≥ 4 weeks oral corticosteroid (OCS) treatment or between clinic visits; or positive bronchial challenge with either PC_20_ of <8mg/ml with methacholine or histamine, or PD_15_ of <635mg with mannitol. Finally, participants were required to have difficult-to-treat asthma as per SIGN/BTS guidelines [2] with persistent or uncontrolled asthma symptoms (defined as a score of ≥1.5 on the Asthma Control Questionnaire (ACQ6)) and/or recurrent exacerbations (≥ two OCS courses or ≥ 1 hospitalization with asthma in the preceding 12 months) despite high dose inhaled corticosteroids (ICS)/long-acting beta agonist (LABA) combination therapy or medium- dose ICS/LABA and oral agent (e.g., leukotriene receptor antagonists, theophylline).

Exclusion criteria included intensive care asthma admission in the preceding 6 months, respiratory tract infection or asthma exacerbation requiring treatment in the preceding 4 weeks, significant co-morbidity likely to influence study conduct, pregnancy or breast feeding, commencement of biologic in the preceding 6 months, or current use of either insulin or anti-obesity medications.

Participants not wishing to continue intervention were invited to continue attending study visits for data collection.

Recruitment was halted early after a greater number than expected of participants adhered successfully to the 12-weeks intensive part of the intervention. Following that, retention was reliably expected to follow a similar pattern to that in the larger DiRECT trial, upon which this trial was based.

*Measurements*

Anthropomorphic measurements – height, weight, BMI, waist circumference, hip circumference, waist-to-height and waist-to-hip ratios.

Height was measured in centimetres using a portable stadiometer, seca 213 (seca, Hamburg, Germany, 2018), to 1mm accuracy. Participants were asked to remove footwear and stand upright and straight prior to measuring. Weight was measured in kilograms using electronic scales, Charder MS4202L (Charder Medical, Taichung City, Taiwan, 2014), to 100g accuracy. Participants were asked to remove outer layers of clothing, footwear, and heavy items in pockets prior to measuring. These measuring devices have been calibrated and certified for use.

Presence of mental health problems was defined by participant self-report and by review of the electronic health record. Atopy was defined by presence on the electronic health record of either a positive IgE to a specific allergen or a positive skin prick test. Osteopenia/osteoporosis were confirmed from review of bone densitometry results on the electronic health record.

Healthcare usage – number of prednisolone courses, out-of-hours General Practice (GP) attendances, Emergency Department (ED) attendances, hospital admissions and Intensive Care Unit (ICU) admissions in the last 12 months or, at follow-up visits, since the last study visit. These variables were self-reported by participants and, where possible, confirmed with the participant electronic health record. For Visit 2 these variables were converted to “annualised” numbers, i.e., (number of events x 365)/number of days between visits.

Blood sampling - full blood count (including eosinophils), urea and electrolytes, liver function tests, bone profile, glucose, insulin, glycated haemoglobin, lipid profile, c-reactive protein, leptin, adiponectin and interleukin (IL)-6.

Physical activity data was collected using an ActiGraph wGT3X-BT (ActiGraph, Pensacola, USA) accelerometer device worn on the non-dominant wrist for 24 hours, 7 days (except when bathing or swimming). Each accelerometer was initialized to capture data at 30 Hz and programmed to commence data collection immediately after distribution. After 7 days, participants returned their accelerometer device with data downloaded using ActiLife software (v.6.14.3; ActiGraph) and saved in raw format as .gt3x files. These files were then converted to .csv files and exported into R v4.1.2 (R Foundation for Statistical Computing, Vienna, Austria) for subsequent processing using the GGIR package (v2.6.0). Briefly, the package detected non-wear time, abnormally high values and auto-calibrated raw triaxial accelerometer signals using local gravity as reference [3]. It then calculated Euclidean Norm Minus One (ENMO) (1 *g*) averaged over 5-second epochs, expressed in milli-gravitational (m*g*) units [4]. Thereafter, inactive time was defined as time accumulated below an acceleration of 30 m*g,* with light (LPA) defined as time spent between 30–99 m*g* [5]. MVPA was defined as time accumulated ≥100 m*g* [6]. Files were excluded from subsequent analyses if post-calibration error was >0.01 g, there were <4 days (defined as ≥16 hours per day) of valid wear including at least 1 weekend day, or wear data was not present for each 15-minute period of the 24-h cycle. The non-wear setting was used [7] meaning outcome variables were calculated based on the entire 24-h cycle.

*Counterweight-Plus weight management programme*

TDR phase - Ample fluid intake was encouraged, and a soluble fibre supplement was provided. Oral hypoglycaemics, antihypertensives and diuretic drugs were withdrawn on commencement of TDR and reintroduced according to national clinical guidelines if hypertension or diabetes recurred. Aspirin and beta-blocker use for ischaemic heart disease was permitted to continue, though aspirin solely prescribed for diabetes mellitus was discontinued.

Food re-introduction phase: week 13 - LELD of approximately 400 kcal/day with the addition of one low-fat meal (c. 360-400 kcal/day), 2 fruit portions, 200mls skimmed milk and free-use of vegetables (totalling 1000 kcal/day; approximately 50% carbohydrate, 35% total fat, and 15% protein); week 15 - LELD of 200 kcal/day, two low-fat meals, alongside fruit, milk and vegetables as above (1200 kcal/day); week 17 – 3 low-fat meals and fruit, milk and vegetables as above (1400 kcal/day).

*Other outcomes (over 16 weeks, between groups)*

Exacerbations: number of courses of high-dose prednisolone for asthma; out-of-hours GP attendances; emergency department admissions; hospital admissions; ICU admissions.

Weight, BMI, waist-to-height and waist-to-hip change.

MRC dyspnoea score, 6MWD, HAD scores, FEV_1_ and FVC change.

*Statistical analysis*

Due to the effects of the COVID-19 pandemic, there were several variables at Visit 2 with large proportions of missing data, those requiring physical attendance (spirometry, blood sampling, FeNO, 6MWT and accelerometery). This was a direct effect of national lockdowns and therefore missing completely at random (MCAR). As a result, they were analysed on a complete case analysis basis and not appropriate for missing data handling methods such as multiple imputations. None of these variables include the primary or key secondary variables (which were able to be collected virtually) however, and all have been reported. This has been highlighted in the limitations section.

**Results**

*Participation*

From the 36 participants screened, 1 was ineligible due to privately starting anti-obesity medication since being approached for the trial, and 2 were lost to follow-up as they did not attend Visit 2 (33 for intention to treat analysis). For per-protocol analysis, 2 participants were excluded as they did not continue CWP intervention (1 was unable to give up food despite encouragement and support, and the other found the regime difficult whilst caring for a family member).

**Supplementary tables**

**Table E1: Supplementary baseline characteristics**

| **Variable** | **Overall n = 35** | **CWP group n = 18** | **UC group n = 17** |
| --- | --- | --- | --- |
| Waist circumference (cm)  Hip circumference (cm) | 121.0 (116.5, 125.4)  123.0 (117.4, 128.7) | 122.8 (117.3, 128.3)  126.7 (121.4, 132.0) | 119.1 (111.5, 126.7)  119.1 (108.6, 129.6) |
| Waist-to-hip ratio  Waist-to-height ratio | 0.99 (0.96, 1.02)  0.74 (0.71, 0.77) | 0.97 (0.93, 1.01)  0.76 (0.71, 0.80) | 1.01 (0.97, 1.05)  0.72 (0.68, 0.77) |
| Borg score | 3 (3, 4) | 3 (2, 4) | 3 (3, 4) |
| Accelerometery (min.d^-1^):  Inactive time  Time in LPA  Time in MVPA | 1164 (1115, 1212)  227 (187, 266)  37.3 (22.5 to 79.7) | 1211 (1159, 1263)  189 (149, 229)  26.8 (12.3 to 75.3) | 1108 (1026, 1190)  271 (200, 341)  61.0 (36.7 to 86.1) |
| Continuous variables described as mean (95% confidence intervals) if parametric or median (first quartile to third quartile) if non-parametric.  Categorical variables described as no. (%).  Abbreviations: CWP (Counterweight Plus); LPA (Low Physical Activity); MVPA (Moderate to Vigorous Physical Activity); UC (Usual Care). | | | |

**Table E2: Between-visit comparison of 16-week asthma control and quality of life outcomes in Counterweight Plus (CWP) group against usual care (UC)**

| **Variable** | **CWP group (n = 17)** | **UC group (n = 16)** | **p-value** |
| --- | --- | --- | --- |
| ACQ6: Visit 1  Visit 2  Change | 2.6 (2.1, 3.2)  2.2 (1.5, 2.9)  -0.4 (-1.0, 0.1) | 2.7 (2.1, 3.3)  2.9 (2.2, 3.6)  0.2 (-0.2, 0.6) | 0.837  0.111  **0.050** |
| AQLQ: Visit 1  Visit 2  Change | 3.9 (3.4, 4.5)  4.7 (4.1, 5.3)  0.8 (0.3, 1.3) | 3.8 (3.2, 4.5)  3.9 (3.4, 4.5)  0.1 (-0.3, 0.5) | 0.867  **0.043**  **0.028** |
| AQLQ Symptom: Visit 1  Visit 2  Change | 3.8 (3.3, 4.4)  4.8 (4.2, 5.5)  1.0 (0.4, 1.5) | 3.9 (3.2, 4.6)  4.1 (3.6, 4.7)  0.3 (-0.1, 0.6) | 0.948  0.093  **0.027** |
| AQLQ Activity: Visit 1  Visit 2  Change | 4.0 (3.4, 4.5)  4.5 (3.8, 5.1)  0.5 (0.0, 1.1) | 3.7 (3.0, 4.4)  3.5 (2.9, 4.1)  -0.1 (-0.7, 0.5) | 0.493  **0.029**  0.085 |
| AQLQ Emotional: Visit 1  Visit 2  Change | 3.7 (2.8, 4.6)  5.2 (4.4, 6.0)  1.5 (0.6, 2.3) | 3.9 (3.1, 4.8)  4.6 (3.8, 5.3)  0.7 (0.1, 1.3) | 0.743  0.248  0.119 |
| AQLQ Environmental: Visit 1  Visit 2  Change | 4.1 (3.4, 4.7)  4.6 (3.9, 5.3)  0.5 (-0.3, 1.3) | 4.2 (3.4, 5.0)  3.7 (2.8, 4.6)  -0.5 (-1.3, 0.3) | 0.792  0.100  0.053 |
| Continuous variables described as mean (95% CI).  p value compares CWP vs UC using unpaired t test.  Abbreviations: ACQ6 (Asthma Control Questionnaire); AQLQ (Asthma Quality of Life Questionnaire); CWP (Counterweight Plus); UC (Usual Care). | | | |

**Table E3: Proportion of intention-to-treat participants achieving MCID in asthma control and quality of life scores**

| **Variable** | **CWP group (n = 17)** | **UC group (n = 16)** | **p-value** |
| --- | --- | --- | --- |
| ACQ6 MCID change | 9 (52.9) | 3 (18.8) | **0.041** |
| AQLQ MCID change | 10 (58.8) | 5 (31.3) | 0.112 |
| AQLQ symptoms MCID change | 12 (70.6) | 7 (43.8) | 0.119 |
| AQLQ activity MCID change | 8 (47.1) | 4 (25.0) | 0.188 |
| AQLQ emotional MCID change | 12 (70.6) | 12 (75.0) | 1.000 |
| AQLQ environmental MCID change | 10 (58.8) | 5 (31.3) | 0.112 |
| Variables described as number (%) and compared using chi-square or Fisher’s exact as appropriate.  Abbreviations: ACQ6 (Asthma Control Questionnaire); AQLQ (Asthma Quality of Life Questionnaire); CWP (Counterweight Plus); MCID (Minimal Clinically Important Difference); UC (Usual Care). | | | |

**Table E4: Supplementary intention-to-treat comparison of outcomes between CWP and UC**

| **Change in variable** | **CWP group** | | **UC group** | | **Mean difference between CWP and UC** | **p-value*** |  |
| --- | --- | --- | --- | --- | --- | --- | --- |
|  | **N** | **Difference** | **N** | **Difference** |  |  |  |
| Waist circumference (cm)  Hip circumference (cm) | 7  7 | -10.9 (-18.5, -3.3)  -8.2 (-16.5, 0.2) | 6  6 | -1.7 (-7.4, 4.1)  5.3 (0.2, 10.4) | -9.3 (-18.0, -0.6)  -13.5 (-22.5, -4.4) | **0.039**  **0.007** |  |
| Waist-to-height ratio  Waist-to-hip ratio | 7  7 | -0.07 (-0.12, -0.02)  -0.03 (-0.09, 0.03) | 6  6 | -0.01 (-0.04, 0.02)  -0.06 (-0.12, 0.00) | -0.06 (-0.11, -0.01)  0.03 (-0.04, 0.11) | **0.029**  0.318 |  |
| Borg score | 8 | -1 (-2 to 0) | 5 | -1 (-3 to 0) | n/a | 0.724 |  |
| Accelerometery (min.d^-1^):  Inactive time  Time in LPA  Time in MVPA | 5  5  5 | -38.1 (-89.1, 12.9)  29.0 (-11.2, 69.2)  9.1 (-23.0, 41.2) | 3  3  3 | 8.3 (-80.9, 97.6)  -9.6 (-84.3, 65.1)  1.3 (-13.4, 16.0) | -46.4 (-116.9, 24.1)  38.6 (-17.9, 95.1)  7.8 (-30.4, 46.0) | 0.158  0.145  0.635 |  |
| Continuous variables described as mean (95% confidence intervals) if parametric or median (first quartile to third quartile) if non-parametric.  *Comparison using independent t test for parametric or Mann Whitney U test for non-parametric data.  Abbreviations: CWP (Counterweight Plus); LPA (Low Physical Activity); MVPA (Moderate to Vigorous Physical Activity); UC (Usual Care). | | | | | | | |

**Table E5: Per protocol comparison of 16-week asthma outcomes between CWP and UC**

| **Change in variable** | **CWP group (n = 15)** | **UC group (n = 16)** | **Mean difference between CWP and UC** | **p-value*** |
| --- | --- | --- | --- | --- |
| ACQ6 | -0.60 (-1.20, 0.01) | 0.23 (-0.17, 0.63) | -0.86 (-1.55, -0.18) | **0.015** |
| AQLQ | 0.97 (0.42, 1.53) | 0.08 (-0.32, 0.48) | 0.95 (0.40, 1.50) | **0.001** |
| AQLQ Symptom | 1.11 (0.55, 1.68) | 0.25 (-0.13, 0.63) | 0.89 (0.32, 1.46) | **0.003** |
| AQLQ Activity | 0.72 (0.25, 1.19) | -0.13 (0.73, 0.46) | 0.97 (0.32, 1.62) | **0.005** |
| AQLQ Emotional | 1.45 (0.45, 2.46) | 0.66 (0.07, 1.25) | 0.85 (-0.62, 1.75) | 0.067 |
| AQLQ Environmental | 0.66 (-0.17, 1.49) | -0.52 (-1.30, 0.26) | 1.18 (0.21, 2.14) | **0.018** |
| Variables described as mean (95% confidence intervals).  *Comparison of mean difference using ANCOVA with baseline variable as covariate.  Abbreviations: ACQ6 (Asthma Control Questionnaire); AQLQ (Asthma Quality of Life Questionnaire); CWP (Counterweight Plus); UC (Usual Care). | | | | |

**Table E6: Per protocol comparison of other outcomes between CWP and UC**

| **Change in variable** | **CWP group** | | **UC group** | | **Mean difference between CWP and UC** | **p-value*** |
| --- | --- | --- | --- | --- | --- | --- |
|  | **N** | **Difference** | **N** | **Difference** |  |  |
| Weight (kg)  Total body weight (%) | 12  12 | -14.7 (-18.0, -11.4)  -13.3 (-16.2, -10.5) | 9  9 | -1.4 (-3.2, 0.4)  -1.2 (-3.0, 0.7) | -13.3 (-17.2, -9.4)  -12.2 (-15.6, -8.8) | **<0.001**  **<0.001** |
| BMI (kg/m^2^) | 12 | -5.3 (-6.4, -4.1) | 9 | -0.3 (-1.1, 0.6) | -5.0 (-6.5, -3.5) | **<0.001** |
| Waist circumference (cm)  Hip circumference (cm) | 6  6 | -12.4 (-20.7, -4.1)  -10.5 (-18.0, -3.1) | 6  6 | -1.7 (-7.4, 4.1)  5.3 (0.2, 10.4) | -10.8 (-19.5, -2.0)  -15.8 (-23.6, -8.0) | **0.021**  **0.001** |
| Waist-to-height ratio  Waist-to-hip ratio | 6  6 | -0.08 (-0.13, -0.02)  -0.02 (-0.09, 0.05) | 6  6 | -0.01 (-0.04, 0.02)  -0.06 (-0.12, 0.00) | -0.07 (-0.12, -0.02)  0.04 (-0.04, 0.12) | **0.017**  0.280 |
| MRC dyspnoea scale | 14 | -1 (-1 to 0) | 15 | 0 (0 to 0) | **n/a** | **0.002** |
| HAD:  Anxiety  Depression | 15  15 | 0 (-2, 2)  -2 (-4, 1) | 16  16 | 1 (-1, 2)  1 (-1, 2) | -1 (-3, 2)  -2 (-5, 1) | 0.567  0.134 |
| Eosinophils (x10^9^/L) | 7 | 0.02 (0.00 to 0.12) | 6 | 0.00 (-0.23 to 0.12) | n/a | 0.295 |
| FeNO (ppb) | 7 | 0 (-3 to 26) | 6 | -6 (-28 to 18) | n/a | 0.534 |
| PEF (L/min) | 8 | 39 (-23, 101) | 6 | 7 (-36, 49) | 33 (-41, 106) | 0.350 |
| Spirometry:  Pre-BD FEV_1_ (%)  Pre-BD FEV_1_/FVC  Post-BD FEV_1_ (%) | 7  7  7 | 6.9 (-2.9, 16.6)  -1.12 (-2.44, 0.20)  5.0 (-0.8, 10.8) | 6  6  6 | 3.7 (-1.4, 8.8)  1.09 (-4.25, 6.43)  4.2 (-7.3, 15.7) | 3.2 (-7.1, 13.5)  -2.20 (-7.53, 3.12)  0.8 (-9.9, 11.5) | 0.510  0.346  0.867 |
| Annualised healthcare use:  Prednisolone courses  OOH GP attendances  ED attendances  Hospital admissions  ICU admissions | 15  15  15  15  15 | -2 (-2 to 0)  0 (0 to 3)  0 (0 to 0)  0 (0 to 0)  0 (0 to 0) | 16  16  16  16  16 | -2 (-3 to 1)  0 (0 to 3)  0 (0 to 0)  0 (-1 to 0)  0 (0 to 0) | n/a | 0.861  0.806  0.572  0.379  1.000 |
| 6MWD (m) | 7 | 9 (-20, 37) | 5 | 0 (-50, 50) | 9 (-37, 54) | 0.681 |
| Borg score | 7 | -1 (-2, -1) | 5 | -1 (-3 to 0) | n/a | 1.000 |
| Accelerometery data, min.d^-1^:  Inactive time  Time in LPA  Time in MVPA | 4  4  4 | -40.1 (-115.2, 34.9)  31.5 (-26.9, 90.1)  8.6 (-38.8, 56.0) | 3  3  3 | 8.3 (-80.9, 97.6)  -9.6 (-84.3, 65.1)  1.3 (-13.4, 16.0) | -48.5 (-132.9, 36.0)  41.2 (-26.1, 108.4)  7.3 (-38.6, 53.2) | 0.200  0.176  0.699 |
| Continuous variables described as mean (95% confidence intervals) if parametric or median (first quartile to third quartile) if non-parametric.  *Comparison using independent t test for parametric or Mann Whitney U test for non-parametric data.  Annualised healthcare use variables compare change from baseline data (number of events in prior 12 months) to 16-weeks ([number of events x 365]/number of days between visits).  Abbreviations: BD (Bronchodilator); BMI (Body Mass Index); CWP (Counterweight Plus); ED (Emergency Department); FeNO (Fractional exhaled Nitric Oxide); FEV_1_ (Forced Expiratory Volume in 1 second); FVC (Forced Vital Capacity); HAD (Hospital Anxiety and Depression scale); ICU (Intensive Care Unit); LPA (Low Physical Activity); MRC (Medical Research Council); MVPA (Moderate to Vigorous Physical Activity); OOH (Out-of-hours); PEF (Peak Expiratory Flow); ppb (parts per billion); UC (Usual Care); 6MWD (6 minute Walk Distance). | | | | | | |

**Table E7: Proportion of per protocol participants achieving MCID in asthma control and quality of life scores**

| **Variable** | **CWP group (n = 15)** | **UC group (n = 16)** | **p-value** |
| --- | --- | --- | --- |
| ACQ6 MCID change | 9 (60.0) | 3 (18.8) | **0.018** |
| AQLQ MCID change | 10 (66.7) | 5 (31.3) | **0.049** |
| AQLQ symptoms MCID change | 11 (73.3) | 7 (43.8) | 0.095 |
| AQLQ activity MCID change | 8 (53.3) | 4 (25.0) | 0.106 |
| AQLQ emotional MCID change | 10 (66.7) | 12 (75.0) | 0.704 |
| AQLQ environmental MCID change | 9 (60.0) | 5 (31.3) | 0.108 |
| Variables described as number (%) and compared using chi-square or Fisher’s exact as appropriate.  Abbreviations: ACQ6 (Asthma Control Questionnaire); AQLQ (Asthma Quality of Life Questionnaire); CWP (Counterweight Plus); MCID (Minimal Clinically Important Difference); UC (Usual Care). | | | |

**References**

1. *Global Initiative for Asthma. Global Strategy for Asthma Management and Prevention*. 2015 23/05/2022]; Available from: [www.ginasthma.org](file:///Users/shar6580/Desktop/CHEST%20primary%20paper/www.ginasthma.org).

2. *British Thoracic Society and Scottish intercollegiate guidelines Network. British guideline on the management of asthma. A national clinical guideline.* 2014 23/05/2022]; Available from: <https://www.brit-thoracic.org.uk/quality-improvement/guidelines/asthma/>.

3. Migueles, J.H., A.V. Rowlands, F. Huber, et al., *GGIR: A Research Community–Driven Open Source R Package for Generating Physical Activity and Sleep Outcomes From Multi-Day Raw Accelerometer Data.* Journal for the Measurement of Physical Behaviour, 2019. **2**(3): p. 188-196.

4. van Hees, V.T., Z. Fang, J. Langford, et al., *Autocalibration of accelerometer data for free-living physical activity assessment using local gravity and temperature: an evaluation on four continents.* J Appl Physiol (1985), 2014. **117**(7): p. 738-44.

5. Bakrania, K., T. Yates, A.V. Rowlands, et al., *Intensity Thresholds on Raw Acceleration Data: Euclidean Norm Minus One (ENMO) and Mean Amplitude Deviation (MAD) Approaches.* PLoS One, 2016. **11**(10): p. e0164045.

6. Hildebrand, M., V.A.N.H. VT, B.H. Hansen, et al., *Age group comparability of raw accelerometer output from wrist- and hip-worn monitors.* Med Sci Sports Exerc, 2014. **46**(9): p. 1816-24.

7. van Hees, V.T., L. Gorzelniak, E.C. Dean León, et al., *Separating movement and gravity components in an acceleration signal and implications for the assessment of human daily physical activity.* PLoS One, 2013. **8**(4): p. e61691.
